# Refining cytokine signatures: flow cytometry–based differentiation between TB infection and disease

**DOI:** 10.5588/ijtldopen.25.0781

**Published:** 2026-07-13

**Authors:** K. Doležalová, J. Goliáš, M. Ibrahimová, L. Bača, E. Kopecká, I. Hriciková, S. Rajnochová-Bloudíčková, T. Philipp, M. Koziar Vašáková

**Affiliations:** 1 Department of Paediatrics of the First Faculty of Medicine, Thomayer University Hospital, Charles University, Prague, Czech Republic;; 2 Laboratory of Immunology, Thomayer University Hospital, Prague, Czech Republic;; 3 Department of Respiratory Medicine of the First Faculty of Medicine Charles University, Thomayer University Hospital, Prague, Czech Republic;; 4 Transplantation Centre, Nephrology Department, Institute for Clinical and Experimental Medicine, Prague, Czech Republic;; 5 Department of Rheumatology and Physiotherapy, Thomayer University Hospital, Third Faculty of Medicine Charles University, Prague, Czech Republic.

**Keywords:** tuberculosis, immunity, interferon-gamma release assay, IGRA, flow cytometry–based assay

## Abstract

**BACKGROUND:**

Distinguishing TB infection (TBI) from TB disease remains difficult because routine tests, including tuberculin skin test and interferon-gamma release assay (IGRA), cannot separate these states. We assessed a flow cytometry–based assay that measures T-cell cytokine responses after stimulation with *Mycobacterium tuberculosis*.

**METHODS:**

We enrolled 118 IGRA-positive participants, including individuals with TB/TBI. Individuals were grouped by age and disease category into paediatric/adult TB, TBI, and immunocompromised TBI. Peripheral blood lymphocytes were stimulated with ESAT-6/CFP-10 antigens under CD4^+^-focused (TB1) or combined CD4^+^/CD8^+^ (TB2) conditions. Intracellular IL-10, IL-2, and tumour necrosis factor-α (TNF-α) were quantified by flow cytometry. Cytokine levels were compared using Mann–Whitney *U* tests.

**RESULTS:**

Among children, most cytokine responses overlapped between TBI and TB, except TNF-α in CD8^+^ T cells after TB2 stimulation, which was higher in TB. In adults, IL-10 in CD4^+^ T cells was elevated in TBI after TB1 and TB2 stimulation, whereas IL-2 in CD4^+^ T cells was higher in TB after TB2 stimulation. TNF-α did not differentiate groups in adults.

**CONCLUSION:**

Flow-cytometric cytokine profiling may help distinguish TBI from TB, with age-specific markers. TNF-α in CD8^+^ T cells appears informative in children, while IL-10 and IL-2 in CD4^+^ T cells show promise in adults. Further validation is needed.

TB remains the leading infectious cause of morbidity and mortality worldwide, with 10.7 million new cases estimated in 2024.^1^ Although the highest burden persists in low- and middle-income countries, TB also affects low-incidence regions due to migration, political instability, and increasing numbers of immunocompromised individuals.^2–4^ The host immune response to *Mycobacterium tuberculosis* (*M.tb*) is complex and dynamic.^5^ Progression from TB infection (TBI) to active disease is influenced by multiple factors, including bacterial load, host immune status, and environmental conditions. Immunocompromised patients (such as those with HIV infection, solid-organ transplant recipients, or individuals receiving anti–TNF-α therapy or long-term corticosteroids) are particularly vulnerable both to reactivation and to rapid progression of disease.^6–9^ Distinguishing TBI from active TB is critical for appropriate management. However, currently available tools, such as the tuberculin skin test and interferon-gamma release assays (IGRAs), cannot reliably differentiate between these two conditions. This diagnostic gap complicates clinical decision-making, particularly in borderline cases and high-risk populations.

Building on our previous pilot work,^10^ we aimed to validate a novel flow cytometry–based assay designed to bridge this gap. The assay quantifies cytokine production by CD4^+^ and CD8^+^ T cells following stimulation with *M.tb*-specific antigens, specifically measuring intracellular IL-2, IL-10, interferon-gamma (IFN-γ), and tumour necrosis factor-α (TNF-α). Cytokine production was assessed after an 18–20 h antigen stimulation (TB1/TB2 tubes) using intracellular cytokine staining and flow cytometry. Our objective was to determine whether distinct cytokine signatures can discriminate TBI from active TB in both immunocompetent and immunocompromised patients, thereby providing a complementary diagnostic tool to improve TB management. Mechanistically, the assay leverages the two-tube design of modern IGRAs: the TB1 tube contains short ESAT-6 and CFP-10 peptides presented by major histocompatibility complex (MHC) class II, primarily assessing CD4^+^ T-cell responses, whereas the TB2 tube includes longer peptides recognised by both MHC classes I and II, allowing detection of combined CD4^+^/CD8^+^ responses.

## METHODS

This prospective study was conducted in the Czech Republic, a country with a low incidence of TB (in 2024, the Czech Republic reported 455 cases of TB [all forms], resulting in an incidence rate of approximately 4.18 cases per 100,000 population).^11^ Participants included children and adolescents (≤18 years) and adults (19–91 years), recruited at Thomayer University Hospital (TUH) in Prague and the Institute of Clinical and Experimental Medicine (IKEM) from September 2022 to July 2025. TUH, a tertiary care facility and the national referral centre for comprehensive TB diagnostics and management, enrolled both paediatric and adult patients with TBI (Z22.7) or pulmonary TB (A15 bacteriologically confirmed pulmonary TB and A16 bacteriologically non-confirmed pulmonary TB) through its Departments of Pediatrics and Pneumology. Patients with TBI undergoing anti–TNF-α therapy for rheumatological disease were additionally recruited via the Department of Rheumatology and Physiotherapy. IKEM, the largest specialised clinical and research medical institute in the Czech Republic, contributed organ transplant recipients with TBI. The paediatric cohort was larger because TUH is a national referral centre for paediatric TB contact investigations and follow-up, whereas adult recruitment was limited by fewer eligible referrals and fewer adults undergoing the same immunological sampling during the study period. The studied population characteristics are included in the Table.

**Table.** Population characteristics of included subjects.

Group (number total)	Age (years), range (median)	Gender male vs. female	Nationality	Diagnosis ICD-10 A15 vs. A16 vs. Z22.7
Children (58)	0–18 (7)	28 vs. 30	Czech: 21 (36 %)	12 vs. 21 vs. 25
			Ukrainian: 27 (46 %)	
			Vietnamese: 5 (9 %)	
			Others: 5 (9 %)	
Adults (49)	21–91 (40)	27 vs. 22	Czech: 22 (45 %)	28 vs. 0 vs. 21
			Ukrainian: 21(43 %)	
			Vietnamese: 2 (4 %)	
			Others: 4 (8 %)	
Immunocompromised (11)	36–72 (59)	2 vs. 9	Czech: 11 (100 %)	0 vs. 0 vs. 11

ICD-10 = International Classification of Diseases; A15 = bacteriologically confirmed TB; A16 = bacteriologically non-confirmed TB; Z22.7 = carrier of TB infection.

The primary inclusion criterion was evidence of host interaction with *M.tb*, confirmed by a positive IGRA. Active TB disease was defined as pulmonary TB diagnosed clinically and/or microbiologically (A15 bacteriologically confirmed; A16 bacteriologically non-confirmed), while TBI was defined as IGRA positivity without evidence of active disease (Z22.7).

### Diagnostic tests

**Figure 1.**
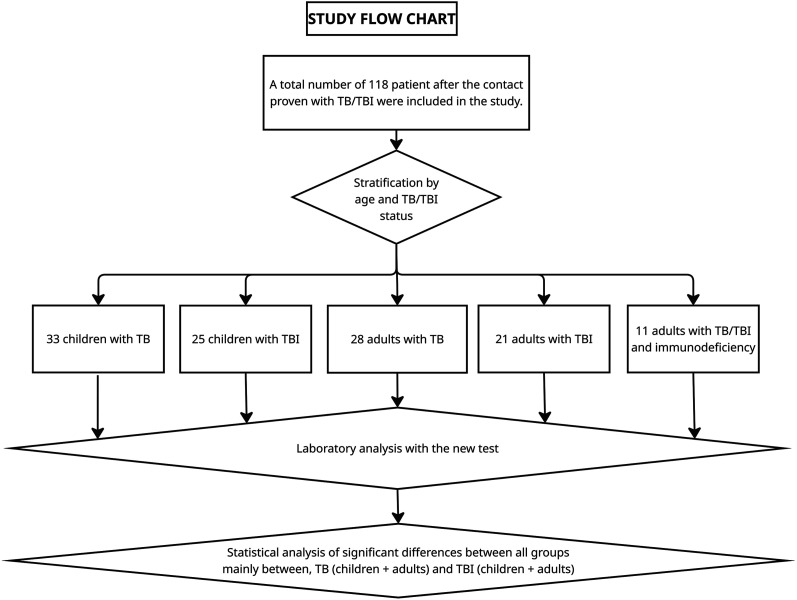
Study flow chart.

A total of 118 individuals with *M.tb* infection confirmed with IGRA test were enrolled for analysis (Figure 1). All participants underwent standard diagnostic evaluation, including microscopy, culture, PCR-based tests, and imaging studies, in accordance with current guidelines. Peripheral blood sampling for IGRA and the flow cytometry assay was performed at baseline, prior to initiation of anti-TB treatment (TB disease) or TB preventive treatment (TBI). Peripheral blood samples were obtained by venipuncture for IGRA (Qiagen, Germany) and for the experimental flow cytometry–based assay. The study cohort was stratified by age group, disease stage (TBI vs TB), and immunocompromised status (e.g., post-transplantation or anti–TNF-α therapy) resulting in 58 children (TBI 25, TB 33), 49 adults (TBI 21, TB 28), and 11 immunocompromised adults (11 TBI). Statistical analyses were then performed to compare immune responses between groups.

This study was designed to assess discrimination between TBI and active TB disease within the IGRA-positive ‘infected spectrum’, which represents the clinically most relevant differential diagnosis once infection has been established. Therefore, we did not include an IGRA-negative/uninfected control group for cytokine analyses. We acknowledge that uninfected controls could further characterise baseline cytokine distributions; however, this was outside the scope of the present validation study and would have required a separate recruitment stream and additional sampling. Uninfected vs infected discrimination using the same platform was evaluated in our pilot work.^10^ Accordingly, cytokine comparisons in the present study were performed between TBI and TB groups.

### Flow cytometric analysis

Peripheral blood was collected and processed in parallel for IGRA and flow cytometric analysis. T cells were stimulated in QuantiFERON-TB Gold Plus TB1 and TB2 tubes (CD4-biased and combined CD4/CD8 stimulation, respectively), with Nil and mitogen tubes as controls. After overnight incubation (18–20 h), Brefeldin A was added to block cytokine secretion, followed by fixation, permeabilisation, and intracellular staining. Cytokine-producing CD4^+^ and CD8^+^ T cells (IL-2, TNF-α, IFN-γ, IL-10) were quantified by multicolour flow cytometry (DxFlex, Beckman Coulter). At least 30,000 CD3^+^ T cells were acquired per sample. Absolute counts were calculated using internal counting beads. Data were analysed using the gating strategy described in our pilot study^10^ with the addition of IL-10. Technical details, antibody clones, and gating strategy are provided in the Supplementary Data.

### Statistical analysis

Cytokine responses were analysed as absolute counts, computed from cytokine-positive percentages and corrected by subtracting Nil controls (negative values set to zero). Sixteen immune readouts (IL-2, IL-10, TNF-α, IFN-γ × TB1/TB2 × CD4/CD8) were evaluated separately in children and adults. Results are presented as medians (interquartile range). Group differences between TB and TBI were assessed using two-sided Mann–Whitney *U* test; *P* values were not corrected due to the exploratory design. A subgroup of TBI immunocompromised adults was compared with immunocompetent adults using the same approach. For adults, a multivariable logistic regression model (12 Δ-variables) was applied to estimate the probability of TB, using thresholds of 0.50 and 0.70. All analyses were performed in R (version 4.4.1). The reproducible analysis script is provided in the Supplementary Data.

### Ethical statement

Written informed consent was obtained from all participants; for minors, consent was provided by legal guardians. The study was approved by the Ethics Committee of the Institute for Clinical and Experimental Medicine and Thomayer Hospital (approval code 16726/24; G-24-17) and conducted in accordance with the Declaration of Helsinki.

## RESULTS

We compared antigen-induced cytokine responses between TBI and active TB disease separately in children and adults; immunocompromised participants were available only in the TBI group.

### Paediatric population

**Figure 2.**
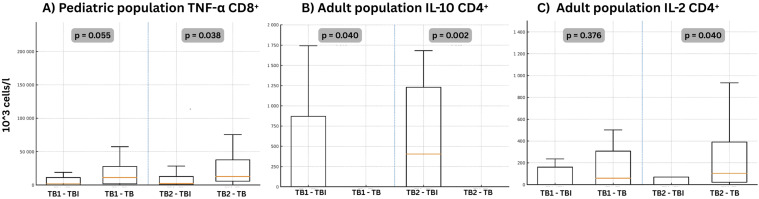
Selected cytokine responses after stimulation in paediatric and adult populations. The y-axis shows the absolute counts of cytokine-producing cells, calculated as: Absolute count = (% positive cells/100) × total cell count. The x-axis represents the cohorts of patients with TB disease (TB) and TB infection (TBI), tested after TB1 and TB2 stimulation. **A:** In the paediatric population, TNF-α production in CD8^+^ cells after TB2 stimulation significantly differentiated TB from TBI (*P* = 0.038). **B:** In the adult population, IL-10 production in CD4^+^ cells showed significant differences after both TB1 (*P* = 0.040) and TB2 (*P* = 0.002) stimulation. **C:** In adults, IL-2 production in CD4^+^ cells after TB2 stimulation was also significantly higher in TB than TBI (*P* = 0.040). Boxes represent interquartile ranges, horizontal lines indicate medians, and whiskers extend to minimum and maximum values.

In children, unstimulated (Nil) samples showed no group differences. After TB1 and TB2 stimulation, cytokine responses increased in both TB and TBI, but only TNF-α in CD8^+^ cells after TB2 reached statistical significance (TBI 2,227.4 [600.0–12,831.6] ×10^3^ cells/L vs. TB 12,765.2 [5,861.5–37,720.8] ×10^3^ cells/L; *P* = 0.038). CD8^+^ TNF-α after TB1 trended toward significance (*P* = 0.055) (Figure 2A). IL-2 and IL-10 responses remained nonsignificant across subsets and conditions. Mitogen stimulation confirmed robust responses in all groups. To conclude, in children, only CD8^+^ TNF-α after TB2 stimulation discriminated TB from TBI (*P* = 0.038); other markers were nonsignificant.

### Adult population

Adults demonstrated higher overall cytokine production than children. Significant differences were observed in CD4^+^ IL-10 and IL-2 responses. TBI participants showed stronger IL-10 responses after TB2 stimulation (TBI 403.9 [0.0–1,229.5] ×10^3^ cells/L vs. TB 0.0 [0.0–0.0] × 10^3^ cells/L; *P* = 0.002) and distributional differences after TB1 (*P* = 0.040) (see Figure 2B). Conversely, TB patients exhibited higher IL-2 responses after TB2 stimulation (TB 104.4 [22.7–390.6] × 10^3^ cells/L vs. TBI 0.0 [0.0–70.2] ×10^3^ cells/L; *P* = 0.040) (Figure 2C). TNF-α responses were largely comparable, with borderline differences in CD8^+^ TB1 responses (*P* = 0.052). To conclude, in adults: TBI was characterised by stronger IL-10 (CD4^+^) responses, whereas TB patients exhibited higher IL-2 (CD4^+^) responses after TB2 stimulation. TNF-α did not reach significance, but in the CD8 subset after TB1 stimulation, the *P* value was 0.052 close to the threshold.

### Immunocompromised patients

The immunocompromised subgroup included only TBI participants. Significant reductions were observed exclusively in TB1-induced cytokine responses. IL-2 production in CD4^+^ T cells was markedly lower compared with immunocompetent adults (median 0.0 [0.0–0.0] vs. 44.9 [0.0–224.6] × 10^3^ cells/L, *P* = 0.018). TB1-induced TNF-α responses were also significantly diminished in both CD4^+^ (0.0 [0.0–801.0] vs. 17,377.2 [0.0–51306.4] × 10^3^ cells/L, *P* = 0.012) and CD8^+^ T cells (0.0 [0.0–110.0] vs. 8,242.0 [0.0–36932.6] × 10^3^ cells/L, *P* = 0.008). No significant differences were observed for TB2 stimulation or for IL-10 and IFN-γ in any T-cell subset (*P* ≥ 0.43) (see Supplementary Data Table S2). Classification using logistic regression yielded distributed probability (P) values: at a P(TB) ≥ 0.50 threshold, 2/11 immunocompromised participants were classified as TB, while at P(TB) ≥ 0.70 only 1/11 remained classified as TB, indicating an absence of strong clustering toward either disease state (Supplementary Data Table S3).

### Discriminant analysis

**Figure 3.**
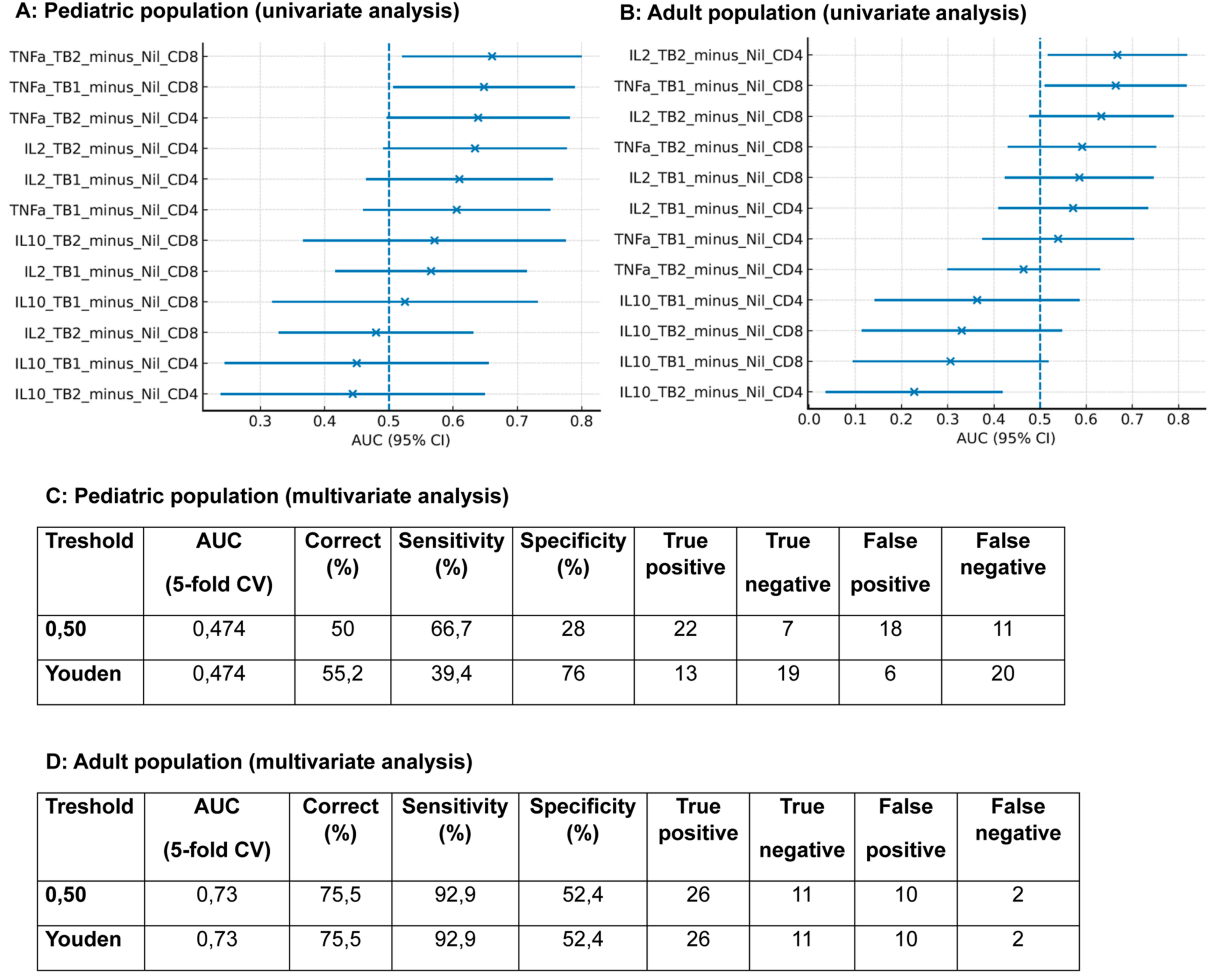
Discriminant analysis between TB and TB infection (TBI) patients: univariate and multivariate approaches. Univariate analyses **(A and B)** assessed 12 cytokine parameters after subtraction of the unstimulated control (Nil). Each forest plot displays AUC values with 95% confidence intervals, with a threshold set at AUC = 0.5. **A:** In the paediatric population, the highest discriminatory performance was observed for TNF-α in CD8^+^ cells following TB1 and TB2 stimulation. **B:** In the adult population, the best discrimination was achieved for IL-2 in CD4^+^ and CD8^+^ cells after TB2 stimulation. Multivariate logistic regression **(C and D)** incorporated all 12 cytokine variables (IL-10, IL-2, and TNF-α responses after TB1/TB2 stimulation in CD4^+^ and CD8^+^ subsets), using absolute cell counts (×10^6^) after Nil subtraction. **C:** In the paediatric cohort, the combined model did not provide clinically useful discrimination (AUC = 0.474, 5-fold CV). **D:** In the adult cohort, the multivariate model showed acceptable performance (AUC = 0.730, 5-fold CV), with overall accuracy 75.5%, sensitivity 92.9%, and specificity 52.4% at a threshold of 0.50. Results remained stable at the Youden threshold (≈0.51), maintaining balanced sensitivity and specificity. AUC = area under the curve; TNF-α = tumour necrosis factor-α.

Discriminant analysis showed only moderate univariate performance in both age groups (area under the curve [AUC] 0.63–0.67). In adults, the multivariate model (all 12 Δ variables) achieved acceptable discrimination (AUC 0.730, 5-fold cross-validation), correctly classifying 75.5% of cases with high sensitivity (92.9%) but lower specificity (52.4%). In children, discrimination was poor (AUC 0.474) (see Figure 3). More detailed results are provided in the Supplementary Data.

## DISCUSSION

Interferon-gamma release assays (IGRAs) revolutionised TB diagnostics by confirming prior exposure to *M.tb*, yet they cannot reliably distinguish TBI from active TB.^12,13^ This gap remains an important unmet need, as such differentiation carries clear clinical implications.

In paediatrics, differentiating TB from TBI in children is especially difficult due to asymptomatic presentations, paucibacillary disease, and nonspecific radiographic findings.^14^ This highlights the need for a more precise test. In our cohort, antigen-specific cytokine responses in children were generally modest. Only CD8^+^ TNF-α production after TB2 stimulation discriminated TB from TBI (*P* = 0.038), with a borderline signal after TB1 stimulation. These findings suggest that paediatric immune signatures may be less polarised during TB progression and that CD8^+^ TNF-α warrants further investigation as a potential marker, though its sensitivity appears limited.

In adults, clinical manifestations are usually clearer, but diagnostic uncertainty persists, particularly in patients with a history of TB, where post-infectious radiographic changes and persistent IGRA positivity complicate interpretation. Our data indicate that adult cytokine profiling may provide additional discriminatory value: TBI was associated with stronger IL-10 responses in CD4^+^ cells (TB2, *P* = 0.002; TB1, *P* = 0.040), while TB patients exhibited higher IL-2 responses after TB2 stimulation (*P* = 0.040). These patterns are consistent with a predominance of regulatory activity (IL-10) in controlled infection and effector activity (IL-2) in active disease. TNF-α responses were less discriminatory, though CD8^+^ TB1 responses approached significance. When combined in a multivariate model, these immune signals produced a discriminant analysis tool that may help in adults as a sensitive screening method: it reduces the risk of missing TB cases, though at the expense of lower specificity.

We further assessed assay performance in immunocompromised adults, a diagnostically challenging population due to attenuated immune responses and the limited reliability of IGRA testing. In this subgroup, TB1-stimulated IL-2 and TNF-α production by both CD4^+^ and CD8^+^ T cells was reduced compared with immunocompetent adults, consistent with diminished antigen-specific activation. However, statistical inference was limited by the small cohort and the absence of participants with active TB. These exploratory findings should therefore be validated in larger, appropriately powered studies. The clinical relevance of more precise immunodiagnostics extends beyond routine differentiation of TB and TBI. In drug-resistant TB, particularly pre-extensively drug-resistant (pre-XDR) or extensively drug-resistant (XDR) disease, preventive treatment of TBI is not indicated, and patients are instead monitored closely.^15^

A tool capable of detecting a shift from controlled infection to active disease could greatly improve management in such cases. Similarly, in low-incidence settings, TB screening before initiation of anti–TNF-α therapy is a frequent clinical dilemma. Patients may present with isolated IGRA positivity, no TB contact history, negative imaging, and negative tuberculin skin test.^16,17^ We have observed cases where IGRA positivity may reflect systemic inflammation rather than true TBI, with conversion to negative after inflammatory control. In such scenarios, a more robust assay could prevent unnecessary TB treatment and delays in anti–TNF-α therapy. Furthermore, anti–TNF-α users and immunocompromised individuals (e.g., those on long-term corticosteroids, with primary or secondary immunodeficiencies) often generate altered or unreliable IGRA results, reinforcing the need for complementary diagnostic approaches.

We built our research on our pilot study in which we used flow cytometry assay incorporating IL-2, TNF-α, and IFN-γ in a TB diagnostics context.^10^ As well, other studies using flow cytometry have demonstrated that *M.tb*-specific T cells can be functionally profiled by their cytokine production to help discriminate against TBI/TB. For instance, Sauzullo et al.^18^ used intracellular cytokine staining for IFN-γ, IL-2, and TNF-α in multi-functional CD4^+^ T cells to explore their diagnostic potential. Maggioli et al.^19^ showed that robust IFN-γ/TNF-α responses by central memory T cells correlate with disease burden, whereas polyfunctional responses including IL-2 may mark a more controlled state. Tebruegge et al.^20^ reported that single-positive TNF-α^+^ and double-positive IFN-γ^+^/TNF-α^+^ CD4^+^ T cells are enriched in TB compared to TBI. Petruccioli et al.^21^ also emphasised that earlier TB flow-cytometry work largely focused on IFN-γ/IL-2 dyads and called for inclusion of TNF-α in polyfunctional analyses. These cumulative efforts justify our focus on that minimal cytokine panel while striving for both biological relevance and translational simplicity.

## CONCLUSION

Although our study did not identify a universally reliable cytokine signature to differentiate TBI from active disease, it revealed distinct immune response patterns that warrant further investigation. In children, CD8^+^ T cell TNF-α responses showed discriminatory potential, while in adults, divergent IL-10 and IL-2 profiles within CD4^+^ subsets appeared more informative. These findings are consistent with previous evidence indicating that IL-2–dominant responses characterise controlled infection, whereas IFN-γ and TNF-α skewing marks active disease, with IL-10 acting as a regulatory counterbalance. By standardising a four-cytokine flow-cytometric panel (IL-2, IL-10, TNF-α, and IFN-γ), our approach contributes to the growing effort to establish reproducible, clinically relevant biosignatures of TB activity. Future research should focus on validating this framework in larger, prospective, and geographically diverse cohorts, expanding cytokine coverage, and exploring integration with molecular and clinical parameters. Ultimately, combining immunological profiling with conventional microbiological diagnostics could enhance early detection, guide treatment selection, and minimise unnecessary drug exposure, particularly in vulnerable paediatric and immunocompromised populations.

## Supplementary Material
