# Supplementary material for: Refining cytokine signatures: flow cytometry–based differentiation between TB infection and disease
Source: IJTLD Open. 2026 Jul 13;3(7):421–7. doi: 10.5588/ijtldopen.25.0781 (PMC13362205; doi:10.5588/ijtldopen.25.0781)
Supplement: Supplementary file 1 [file ijtldopen25-0781_supplementarydata1.pdf]

Supplements

Materials and Methods

Flow cytometric analysis

The flow cytometry assay was developed as a combined approach integrating the principle of interferon-gamma release assays (IGRAs) with flow cytometric detection of intracellular cytokines. We used the TB1 and TB2 antigen tubes from the QuantiFERON-TB Gold Plus system to stimulate T cells: the TB1 tube primarily targets $CD4^+$ lymphocytes, whereas the TB2 tube is designed to activate both $CD4^+$ and $CD8^+$ lymphocytes. Peripheral blood (PB) samples (5 mL) were collected by venipuncture into lithium-heparin tubes (Vacuette, 5 mL, LH Lithium Heparin, Greiner Bio-One, Austria). Of this, 4 mL were used for standard IGRA testing (Qiagen, Germany) following the manufacturer's instructions, and the remaining 1 mL was processed for the experimental flow cytometry assay using the same antigen stimulation tubes.

Whole blood was incubated for 18–20 h in the stimulation tubes—TB1 ($CD4^+$ T-cell response), TB2 (combined $CD4^+$/$CD8^+$ response), and Mit (positive control with phytohemagglutinin, PHA)—alongside an unstimulated Nil tube (negative control). The specific *M. tuberculosis* antigens included ESAT-6 (early secreted antigenic target 6) and CFP-10 (culture filtrate protein 10). The final blood volume per tube was 0.25 mL. After incubation, Brefeldin A (final concentration 10 µg/mL; BioLegend, CA, USA) was added for 4 h to block cytokine secretion. All stimulations were performed at 37 °C in a humidified incubator.

Subsequently, samples were fixed and permeabilized (PerFix-nc kit, Beckman Coulter, France) and stained with surface markers—CD3/AF750 (clone UCHT1), CD4/PB (clone 13B8.2), and CD8/AF700 (clone B9.11)—as well as intracellular cytokine markers—IL-2/PC7 (clone MQ1-17H12), IFN-γ/FITC (clone 45.15), TNF-α/PE (clone IPM2), and IL-10/eFluor 660 (clone JES3-9D7). Staining was performed using DuraClone IF T Activation tubes (Beckman Coulter, India), with the addition of a monoclonal antibody detecting intracellular IL-10 (Fisher Scientific/Invitrogen), according to the manufacturer's recommendations.

Flow cytometric data were acquired using a three-laser DxFlex flow cytometer (Beckman Coulter), enabling detection of IL-2, IL-10, IFN-γ, and TNF-α expression in $CD4^+$ and $CD8^+$ T-cell subsets. For each sample, at least 30,000 $CD3^+$ T cells were collected to ensure sufficient statistical robustness. Absolute cell counts were obtained using Flow-Count Fluorospheres (Beckman Coulter, Ireland). Data were analyzed with the DxFlex and Kaluza C software (Beckman Coulter, CA, USA), following the same gating strategy as in our pilot study, which was described in detail in our previous publication (Tuberculosis 2024), with the addition of IL-10 measurement. PHA stimulation served as a positive control to identify samples with impaired immune responsiveness, reflected by delayed T-cell activation.

Statistical analysis

Quantitative values of IL-10, IL-2, and TNF-α produced by $CD4^+$ and $CD8^+$ T lymphocytes were assessed under four stimulation conditions: Nil (unstimulated), TB1, TB2, and Mitogen (Mit). Analyses were conducted separately for children and adults and stratified by clinical groups (TB/TBI).

Percentages of cytokine-positive cells were converted into absolute counts using the formula:

Absolute count=%positive cells/100×total cell count. Background responses were corrected by subtracting the Nil control from each stimulated condition (TB1, TB2); negative values after subtraction were set to zero.

For each cytokine, age group, and stimulation condition, descriptive statistics were calculated as median with interquartile range (Q1–Q3). Group comparisons (TB/TBI) were performed using the two-sided Mann–Whitney U test (Wilcoxon rank-sum test), chosen as a non-parametric alternative for independent samples. Given the exploratory design, no correction for multiple testing was applied.

A total of 12 outcome variables were analyzed:

- IL-10: ΔTB1_CD4, ΔTB2_CD4, ΔTB1_CD8, ΔTB2_CD8
- IL-2: ΔTB1_CD4, ΔTB2_CD4, ΔTB1_CD8, ΔTB2_CD8
- TNF-α: ΔTB1_CD4, ΔTB2_CD4, ΔTB1_CD8, ΔTB2_CD8

Results are presented as median (Q1–Q3) with corresponding p-values. All analyses were conducted in R (version 4.4.1) using the packages readxl and dplyr for data handling and the base function *wilcox.test* for hypothesis testing.

A subgroup of immunocompromised adults (n = 11, TBI group) was compared with immunocompetent adult controls using two-sided Mann–Whitney U tests. Given the exploratory nature of this analysis, p-values were not adjusted for multiple comparisons. Immune responses were evaluated across 16 Δ variables representing cytokine-expressing $CD4^+$ and $CD8^+$ T cells after stimulation with TB1 or TB2 antigens (IL-2, TNF-α, IL-10, IFN-γ; TB1/TB2 × $CD4^+$/$CD8^+$). All values were calculated as Δ = (stimulated − Nil) and expressed in ×10^3 cells/l; negative Δ values were set to zero.

To explore the diagnostic utility in this subgroup, a multivariable logistic regression model previously trained on immunocompetent adults (12 Δ variables) was applied to estimate the probability of active TB (P(TB)), using decision thresholds of 0.50 and 0.70.

Table 1: Cytokine responses (absolute counts ×10^3 cells/l, median [Q1–Q3]) in TBI vs. TB, stratified by age group

Population	Cytokine	Subset	Stimulation	TBI (median [Q1–Q3])	TB (median [Q1–Q3])	p-value
**Children**	**IL-10**	**CD4$^+$**	TB1	0.0 [0.0–0.0]	0.0 [0.0–0.0]	0.478
		CD4$^+$	TB2	0.0 [0.0–2271.5]	0.0 [0.0–0.0]	0.483
		**CD8$^+$**	TB1	635.8 [0.0-4938.8]	660.0 [172.4-5378.0]	0.825
		CD8$^+$	TB2	385.6 [0.0-21166.0]	2514.4 [1526.0-7103.3]	0.509
	**IL-2**	**CD4$^+$**	TB1	131.7 [0.0-530.6]	269.6 [85.2-666.2]	0.155
		CD4$^+$	TB2	155.4 [0.0-383.6]	425.2 [71.6-895.5]	0.080
		**CD8$^+$**	TB1	0.0 [0.0-331.4]	167.1 [0.0-378.0]	0.379
		CD8$^+$	TB2	137.2 [0.0-403.2]	131.4 [0.0-240.8]	0.796
	**TNF-α**	**CD4$^+$**	TB1	11632.8 [2620.8-35120.4]	25359.5 [11373.0-39495.9]	0.174
		CD4$^+$	TB2	11629.1 [1844.5-30782.4]	27439.8 [10454.5-42844.3]	0.073
		**CD8$^+$**	TB1	1761.2 [0.0-11278.8]	11218.9 [1696.5-27820.8]	0.055
		CD8$^+$	TB2	2227.4 [600.0–12831.6]	12765.2 [5861.5–37720.8]	0.038
	**IFN-g**	**CD4$^+$**	TB1	186.8 [0.0-658.4]	259.0 [0.0-850.4]	0.643
		CD4$^+$	TB2	0.0 [0.0-177.6]	180.2 [0.0-704.7]	0.060
		**CD8$^+$**	TB1	0.0 [0.0-331.4]	33.6 [0.0-270.0]	0.893
		CD8$^+$	TB2	0.0 [0.0-98.8]	0.0 [0.0-285.0]	0.637
**Adults**	**IL-10**	**CD4$^+$**	TB1	0.0 [0.0–870.7]	0.0 [0.0–0.0]	0.040
		CD4$^+$	TB2	403.9 [0.0–1229.5]	0.0 [0.0–0.0]	0.002
		**CD8$^+$**	TB1	4037.0 [256.3-10279.3]	371.2 [31.4-729.6]	0.100
		CD8$^+$	TB2	3080 [183.2-8922.2]	561.0 [0.0-954.8]	0.148
	**IL-2**	**CD4$^+$**	TB1	0.0 [0.0-160.9]	59.6 [0.0-307.4]	0.376
		CD4$^+$	TB2	0.0 [0.0-70.2]	104.4 [22.7-390.6]	0.040
		**CD8$^+$**	TB1	23.3 [0.0-209.2]	95.5 [0.0-401.8]	0.303
		CD8$^+$	TB2	23.3 [0.0-156.9]	234.3 [0.0-606.6]	0.107
	**TNF-α**	**CD4$^+$**	TB1	13363.5 [743.4-47446.5]	20739.1 [0.0-54542.6]	0.646
		CD4$^+$	TB2	211.5 [0.0-27662.4]	646.8 [0.0-14979.8]	0.659
		**CD8$^+$**	TB1	2296.8 [0.0-20920.0]	13775.0 [781.4-62454.2]	0.052
		CD8$^+$	TB2	0.0 [0.0-18148.1]	3562.4 [0.0-18115.7]	0.267
	**IFN-g**	**CD4$^+$**	TB1	90.6 [0.0-286.8]	37.5 [0.0-221.3]	0.278
		CD4$^+$	TB2	197.7 [0.0-459.0]	0.0 [0.0-225.2]	0.190
		**CD8$^+$**	TB1	36.7 [0.0-146.1]	10.3 [0.0-123.1]	0.757
		CD8$^+$	TB2	34.0 [0.0-203.0]	30.8 [0.0-148.9]	0.933

Table 2: Cytokine responses of immunocompromised vs. immunocompetent adults with TBI (absolute counts ×10^3 cells/l, median [Q1–Q3])

Cytokine	Subset	Stimulation	Immunocompromised (median [Q1–Q3])	Immunocompetent (median [Q1–Q3])	p-value
**IL-10**	**CD4⁺**	TB1	354.0 (0.0-2482.0)	639.2 (15.2-4227.8)	0.580
	CD4⁺	TB2	708.0 (153.0-8386.0)	1256.4 (0.0-2956.5)	0.871
	**CD8⁺**	TB1	16.0 (0.0-178.0)	19.0 (0.0-67.7)	0.946
	CD8⁺	TB2	48.0 (0.0-150.5)	30.4 (0.0-159,0)	0.843
**IL-2**	**CD4⁺**	TB1	0.0 (0.0-0.0)	44.9 (0.0-224.6)	0.018
	CD4⁺	TB2	70.0 (0.0-203.0)	53.2 (0.0-375.6)	0,648
	**CD8⁺**	TB1	173.5 (135.0-193.5)	82.6 (0.0-334.4)	0.303
	CD8⁺	TB2	87.5 (0.0-161.3)	114.6 (0.0-495.6)	0.499
**TNF-α**	**CD4⁺**	TB1	0.0 (0.0-801.0)	17377.2 (0.0-51306.4)	0.012
	CD4⁺	TB2	0.0 (0.0-1648.5)	211.5 (0.0-18669.0)	0.258
	**CD8⁺**	TB1	0.0 (0.0-110.0)	8242.0 (0.0-36932.6)	0.008
	CD8⁺	TB2	0.0 (0.0-1976.0)	1017.9 (0.0-18148.1)	0.113
**IFN-g**	**CD4⁺**	TB1	102.0 (0.0-313.0)	83.9 (0.0-236.1)	0.834
	CD4⁺	TB2	0.0 (0.0-371.5)	44.9 (0.0-265.2)	0.758
	**CD8⁺**	TB1	0.0 (0.0-88.0)	35.8 (0.0-131.6)	0.432
	CD8⁺	TB2	0.0 (0.0-187.0)	32.3 (0.0-203.0)	0.465

Table 3: Classification of immunocompromised participants with TBI using logistic regression

No.	Probability of TB [P(TB)]	Classification at thresholds 0.50	Classification at thresholds 0.70
1	0.346	TBI	TBI
2	0.529	TB	TBI
3	0.363	TBI	TBI
4	0.268	TBI	TBI
5	0.379	TBI	TBI
6	0.304	TBI	TBI
7	0.096	TBI	TBI
8	0.447	TBI	TBI
9	0.748	TB	TB
10	0.235	TBI	TBI
11	0.348	TBI	TBI
